# Towards a new osteometric method for sexing ancient cremated human remains. Analysis of Late Bronze Age and Iron Age samples from Italy with gendered grave goods

**DOI:** 10.1371/journal.pone.0209423

**Published:** 2019-01-30

**Authors:** Claudio Cavazzuti, Benedetta Bresadola, Chiara d’Innocenzo, Stella Interlando, Alessandra Sperduti

**Affiliations:** 1 Durham University, Department of Archaeology, Durham, United Kingdom; 2 Museo delle Civiltà, Servizio di Bioarcheologia, Rome, Italy; 3 Università di Roma “La Sapienza”, Dipartimento di Biologia Ambientale, Rome, Italy; 4 Università degli Studi di Napoli “L’Orientale”, Naples, Italy; Seoul National University College of Medicine, REPUBLIC OF KOREA

## Abstract

Sex estimation of human remains is one of the most important research steps for physical anthropologists and archaeologists dealing with funerary contexts and trying to reconstruct the demographic structure of ancient societies. However, it is well known that in the case of cremations sex assessment might be complicated by the destructive/transformative effect of the fire on bones. Osteometric standards built on unburned human remains and contemporary cremated series are often inadequate for the analysis of ancient cremations, and frequently result in a significant number of misclassifications. This work is an attempt to overcome the scarcity of methods that could be applied to pre-proto-historic Italy and serve as methodological comparison for other European contexts. A set of 24 anatomical traits were measured on 124 Bronze Age and Iron Age cremated individuals with clearly engendered grave goods. Assuming gender largely correlated to sex, male and female distributions of each individual trait measured were compared to evaluate sexual dimorphism through inferential statistics and Chaktaborty and Majumder’s index. The discriminatory power of each variable was evaluated by cross-validation tests. Eight variables yielded an accuracy equal to or greater than 80%. Four of these variables also show a similar degree of precision for both sexes. The most diagnostic measurements are from radius, patella, mandible, talus, femur, first metatarsal, lunate and humerus. Overall, the degree of sexual dimorphism and the reliability of estimates obtained from our series are similar to those of a modern cremated sample recorded by Gonçalves and collaborators. Nevertheless, mean values of the male and female distributions in our case study are lower, and the application of the cut-off point calculated from the modern sample to our ancient individuals produces a considerable number of misclassifications. This result confirms the need to build population-specific methods for sexing the cremated remains of ancient individuals.

## Introduction

The practice of cremation emerged in Europe over an extended period, starting from at least the Mesolithic [1,2], and stabilizing in the Neolithic [3], but during the Copper Age–and even more so during the Bell Beaker period–there was a rapid acceleration in its uptake [4]. Cremation cemeteries appear in the Danubian-Carpathian basin and in the Central Mediterranean from the 3rd millennium BC, but the archaeological record shows relatively small funerary areas, [5]. During the Bronze Age, the transition from inhumation to cremation permeates many areas of continental Europe [6,7]; by the end of the second millennium BC, large “urnfields”, including hundreds (and sometimes thousands) of graves, widely representative of the living community, have become predominant [8–12].

In Northern and peninsular Italy, the transition from inhumation to cremation, with some rare exceptions, unfolds over roughly five centuries, from the Middle/Late Bronze Age to the Early Iron Age [8]. In general, from extremely austere practices that excluded most grave goods (particularly weapons), in order to hide the social status of the deceased (i.e. the gender, age, role or rank), the ritual becomes progressively elaborate [13]. From the final phases of the Bronze Age (ca. 1000 BC), cremation burials include a wide range of offerings and grave goods that emphasize the identity and status of the deceased [8,14–16]. Three major obstacles have therefore long inhibited the socio-biological analysis of the urnfields: the overwhelming number of burials, which necessarily requires huge analytical efforts; the fragmentary nature of the human remains; and the ritual dissimulation manifest in most Bronze Age cremation burials, especially those prior to 1000 BC.

Given the difficulty of assessing sex from fragmentary, burnt skeletal remains, for which sexually dimorphic morphological features are often inaccessible, archaeologists often assign gender, which is a proxy of sex, based on grave goods.

Obviously, gender estimation based on grave goods may not correspond to sex since the first is a social construct and the latter is a biological feature. Moreover, those burials without grave goods frequently, therefore, remain undetermined for sex, and the social structures and dynamics of related populations remain, to an extent, unclear.

Our aim is to test new, more objective and reproducible osteometric strategies to facilitate sex estimations in ancient human calcined remains.

Further incentive for the development of new sexing methods comes from recent advances in cremation studies, especially in relation to the determination of demography and human mobility through strontium isotope analysis [17,18]. This field of research urgently requires the creation of a more solid framework within which to explore sexually differentiated patterns.

### Sex estimation of cremated remains

Heat-induced modifications (fragmentation, warping and dimensional changes) of bones strongly affect the applicability of sexing techniques (both morphological and metrical) developed from/for unburned samples. Few experimental studies on contemporary cremations have attained an acceptable degree of reliability (up to 88% of correct diagnoses) for morphological sex assessment based on skull and pelvis features [19]. However, the most informative traits for sexing skeletons can be lost or significantly modified by heat [20–22]. In the archaeological experience, the rate of underrepresentation is usually high: in the Iron Age Pontecagnano-Colucci sample (N = 40), only 20 individuals presented at least one skull indicator and only seven had at least three; the mastoid was observable in eight cases, the glabella in three. For the pelvis, frequency was even lower with only 10 individuals presenting at least one diagnostic element [23]. Since the morphoscopic methods require the observation of several skeletal features, their reliability is strongly reduced in the cremation contexts.

Cremation affects not only morphologies but also has a significant impact on bone size as registered by experimental studies, each with different outcomes [24–33]. Buikstra and Swegle [27] reported less than 6% shrinkage at temperatures higher than 800°C, whereas most studies demonstrate a variable reduction of up to 25% of the original bone size takes on exposure to 700°C and above, depending on the anatomical area [34].

This evidence prompts two important questions: first, whether the effects of heat can affect intra-sample sexual dimorphism; second, the degree to which the application of conventional metric standards to cremated remains is reliable. Most of the studies indicate that when all the bones are burned at the same conditions (such as duration of the process and temperature) the intra-sample sexual dimorphism is maintained [35–44]. Gonçalves and Gonçalves et al [32,33,45] observed significant levels of sexual dimorphism for several postcranial variables in a sample of modern cremated individuals from Portugal. Nevertheless, on the same sample, he recorded very low values of correct sex classifications when using the standard cut-off points recommended by Wasterlain and Cunha and by Silva for unburnt skeletons [46,47]. As might be expected, as consequence of the dimensional changes (mostly referable to the shrinking effect), the misclassification of males exceeds that of female. Misclassifications range from 30.4% using the maximum length of the calcaneus, up to 77.3% using the transverse diameter of the femoral head. Furthermore, these strong differences between variables may be responsible for intra-individual inconsistency in the sex diagnosis. This phenomenon is mostly attributable to differential effects of the cremation process on various skeletal parts, likely linked to their relative position with respect to the heat source, their specific bone structure and anatomy, amount of soft tissues, and presence of personal items [48].

The first attempt to create specific standards for 126 cremated individuals dates to the experimental study of Gejvall [35], who analyzed the degree of sexual dimorphism of seven cranial and infracranial metric variables in a contemporary, cremated sample of known sex. While the approach has proved to be valuable, the Author himself expressed reservations about the extensive use of his data for sexing unknown individuals. Indeed, the inadequacy of Gejvall values in relation to the Italian protohistoric series has been recently demonstrated [49].

Other scholars have investigated the potentiality of osteometry for sex determination of burnt skeletons. Van Vark [37] and Van Vark et al. [42] successfully tested the discriminatory power of several cranial and post-cranial features on a sample of 251 modern North European individuals. Other studies were performed on different samples taking into account specific sets of variables with different outcomes [40,43,50–52]

Promising results were recently obtained on modern Portuguese cremations [32,53]. The Authors developed a set of univariate osteometric standards—on humerus, femur, talus, and calcaneus—achieving successful results in the application of cut-off points and logistic regression equations [53].

The main aim of our own analysis was to test the potential of a large set of metric variables and evaluate their discriminatory power for sex estimation. Samples of cremated remains from five protohistoric Italian necropolises were considered, using the “known gender”, as inferred from the archaeological record, as the proxy for sex. In the absence of census data and written sources for the period, this represents the only viable strategy for building population-specific reference distributions. The results provide a baseline for further analyses on new and old osteological collections.

We are aware of the distinction between sex and gender, whereby the former is defined as a universal biological category and the latter is a cultural/social construction that varies in time and space [54–56]. If the classifying variable is not totally independent and varies with context, it seems likely that, at least in this chronological and geographical framework, sexual identity will coincide with gender in the vast majority of the cases, as testified by a significant correlation between archaeological materials and osteological data, both for cremations and inhumations [57–59].

## Materials

**Fig 1:**
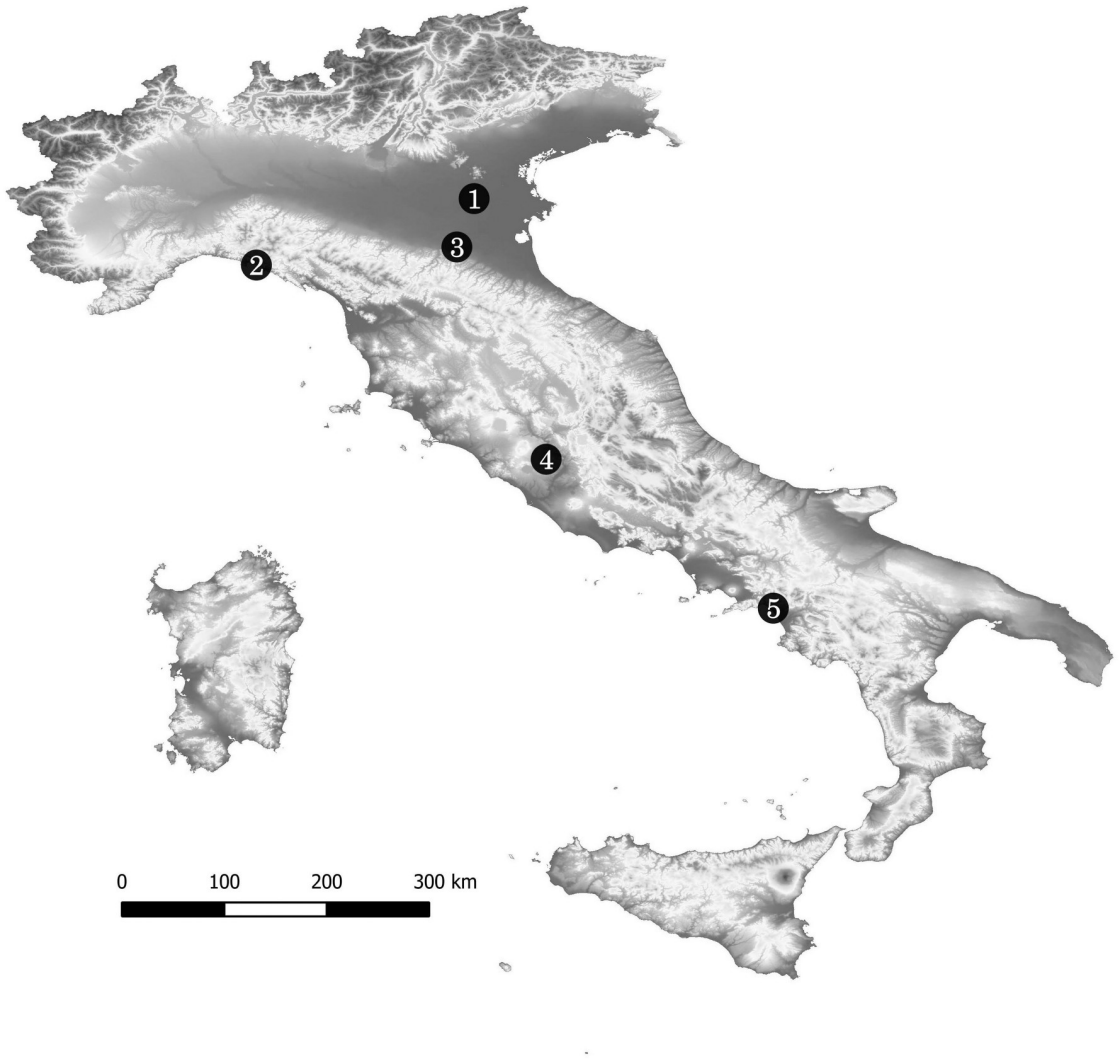
Geographical localization of the sites. 1. Narde di Frattesina (Fratta Polesine, Rovigo); 2. Chiavari (Genova); 3. Castenaso (Bologna); 4. Narce (Faleri, Viterbo); 5. Pontecagnano (Salerno). The map is constructed by using public domain wms data downloadable from http://www.pcn.minambiente.it/mattm/servizio-wms/ under a CC BY license, with permission from Geoportale Nazionale.

Osteometric data were collected for a total of 124 adult individuals from the Final Bronze to the Iron Ages (50 males and 74 females; Table 1; S1 Table), whose remains are held at the Service of Bioarchaeology at the Museo delle Civiltà (P. le Marconi 14, 00144, Rome), where documentation about burials and skeletal materials is also available. According to Italian legislation, no permits were required for the described study, which complied with all relevant regulations. The archaeological sites included in the study were: Narde di Frattesina (burial groups Narde 1 and Narde 2) [16,60], Chiavari [61], Narce [62], Castenaso, Pontecagnano [63] (see Fig 1 and Table 1 for geographical and chronological details respectively). Narde di Frattesina represents the most ancient necropolis (12th–9th century BC), while Chiavari is the most recent (7th–6th century BC).

**Table 1:** Details of the archaeological sites and samples.

*Necropolis*	*Province (Region)*	*Chronology*	*N males*	*N females*	*References*
Narde di Frattesina	Rovigo (Veneto)	Final Bronze Age (12^th^-9^th^ century BCE)	19	40	[60]
Chiavari	Genova (Liguria)	Iron Age (7^th^-6^th^ century BCE)	10	12	[75]
Castenaso, Madonna del Buon Consiglio	Bologna (Emilia Romagna)	Iron Age—Villanovian (7^th^ century BCE)	2	1	Unpublished
Narce	Viterbo (Lazio)	Iron Age—Faliscan (8^th^-7^th^ century BCE)	3	3	[76]
Pontecagnano	Salerno (Campania)	Iron Age (8^th^-7^th^ century BCE)	16	18	[77]

**Fig 2:**
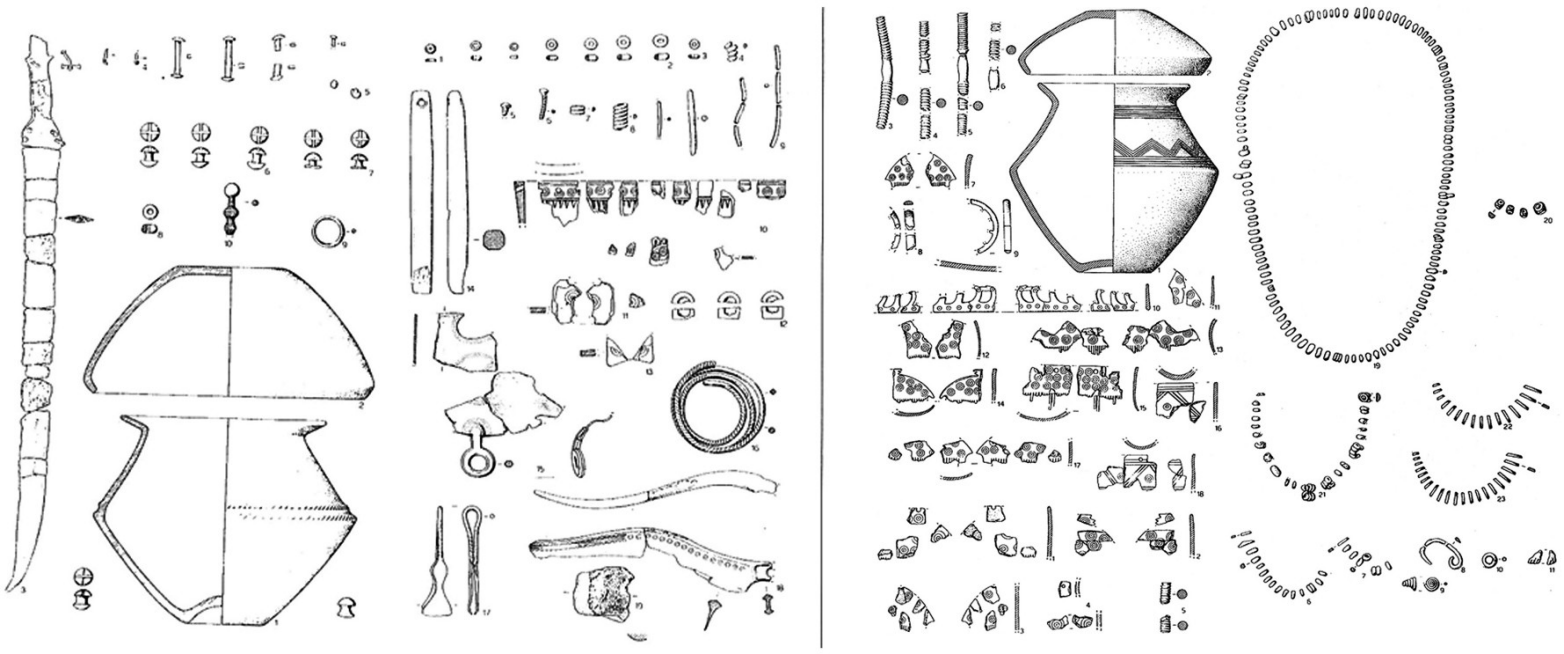
Clearly engendered grave good assemblages. Burials with typical masculine (left) and feminine grave goods (right) from Narde di Frattesina urnfield (mod. after [67]).

The individuals were selected using the following criteria: (1) burials with one single individual, to avoid misleading determinations; (2) only adult individuals (older than 20 years), with fully developed skeletons and all the epiphyses completely fused [64]; (3) bone chromatism ranging from white calcined, to gray, typical of a complete cremation (above 700°C), to restrict the variability of dimensional changes [65,66]; (4) bones free from osteoarthritic alterations or other visible skeletal pathologies; (5) burials including a substantial number and/or quality of gender-specific shaped urns and/or grave goods (weapons and razors for men; spindle whorls, simple-arch or “leech” fibulas, faïence or glass beads for women), providing a strong indication of gender (Fig 2).

## Methods

**Fig 3:**
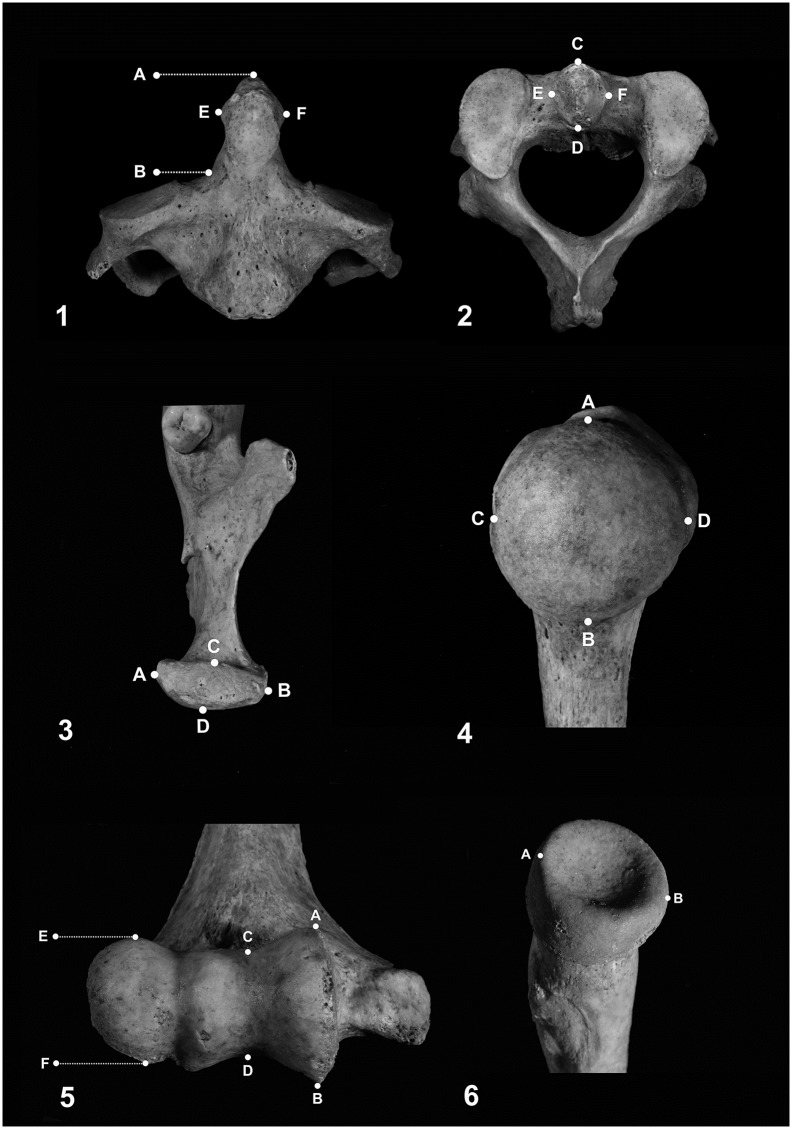
Schematic illustration of 11 measurements used in the study. 1 & 2) Axis: A-B = dens height; C-D = dens anteroposterior diameter; E-F = dens transverse diameter. 3) Mandible: A-B = condyle width; C-D = condyle thickness. 4) Humerus head: A-B = vertical diameter; C-D = transverse diameter. 5) Humerus distal epiphysis: A-B = trochlea maximum diameter; C-D = trochlea minimum diameter; E-F = capitulum maximum diameter. 6) Radius: A-B = head maximum diameter.

**Fig 4:**
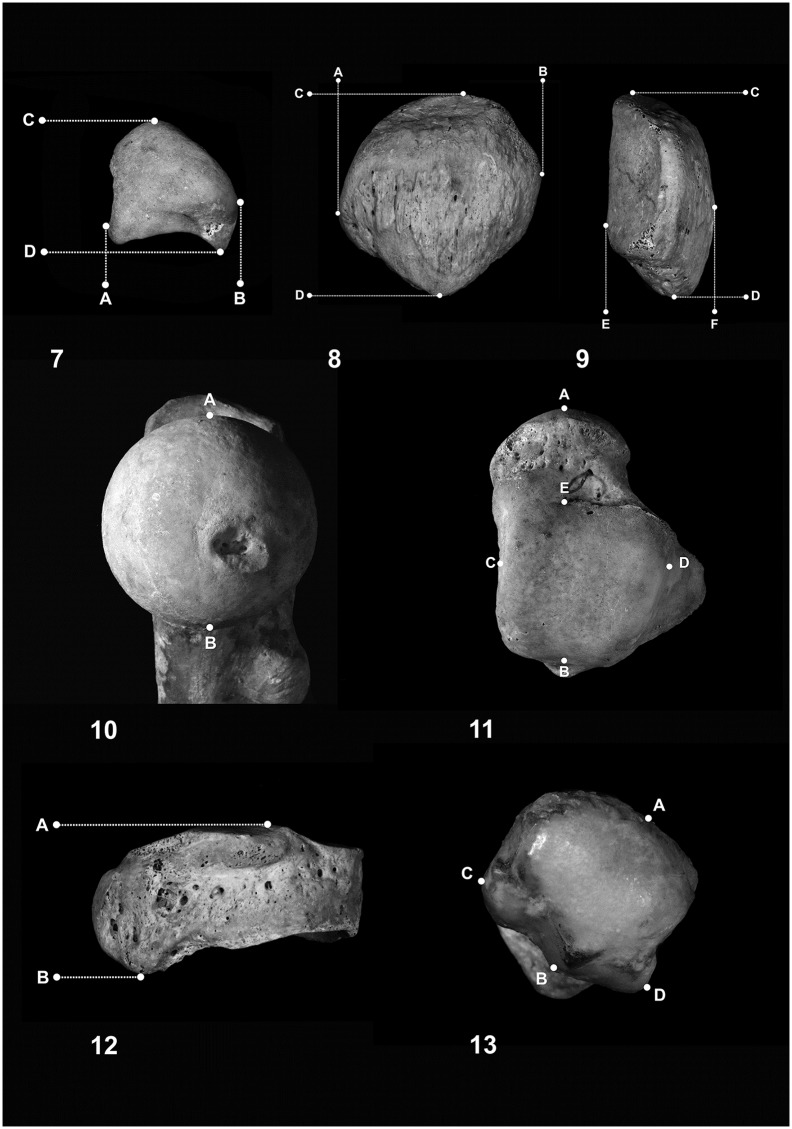
Schematic illustration of 13 measurements used in the study. 7) Lunate: A-B = maximum width; C-D = maximum length; 8 & 9) Patella: A-B = maximum width; C-D = maximum height; E-F maximum thickness; 10) Femural head: A-B = vertical diameter; 11) Talus: A-B = maximum length; A-E = head-neck length; C-D = trochlea width; E-B = trochlea length; 12) Navicular: A-B = maximum length; 13) First metatarsal head: A-B = dorso-plantar width; C-D = medio-lateral width.

The osteometric analysis considered 24 variables (Table 2; Figs 3 and 4). Selection of variables was based on four criteria: (1) they are from skeletal elements that show a high rate of preservation in cremains; (2) they are characterized by easily detectable landmarks; (3) they show a good degree of sexual dimorphism in unburnt skeletons; (4) they were considered in previous studies.

**Table 2:** TEM calculation of the 24 metrical variables considered in this study.

*Trait*	*Abbreviation*	*MEAN (mm)*	*TEM (mm)*	*RTEM (%)*
Mandible: condyle width	MA-C-W	16.16	0.199	1.2
Mandible: condyle thickness	MA-C-TH	6.85	0.348	**5.1**
Axis: dens height	AX-D-H	12.25	1.444	**11.8**
Axis: dens anteroposterior diameter	AX-D-APD	9.50	0.147	1.5
Axis: dens transverse diameter	AX-D-TD	8.70	0.063	0.7
Humerus: head vertical diameter	HU-H-VD	36.99	0.213	0.6
Humerus: head transverse diameter	HU-H-TD	33.45	1.850	**5.5**
Humerus: trochlea maximum diameter	HU-T-MXD	19.83	0.226	1.1
Humerus: trochlea minimum diameter	HU-T-MID	12.79	0.234	1.8
Humerus: capitolum maximum diameter	HU-C-MXD	16.04	0.369	2.3
Radius: head maximum diameter	RD-H-MD	18.15	0.207	1.1
Lunate: maximum width	LU-MXW	13.65	0.110	0.8
Lunate: maximum length	LU-MXL	13.42	0.328	2.4
Femur: head vertical diameter	FE-H-VD	37.70	0.193	0.5
Patella: maximum height	PA-MXH	35.51	0.281	0.8
Patella: maximum width	PA-MXW	35.87	0.232	0.6
Patella: maximum thickness	PA-MXTH	15.35	0.340	2.2
Talus: maximum length	TA-TL	45.74	0.315	0.7
Talus: head-neck length	TA-HN-L	19.23	0.272	1.4
Talus: trochlea length	TA-TR-L	29.87	0.403	1.3
Talus: trochlea width	TA-TR-W	27.20	0.291	1.1
Navicular: maximum length	NA-MXL	12.89	0.477	3.7
First Metatarsal: dorso-plantar width of head	MT1-H-DPW	16.01	0.320	2.0
First Metatarsal: medio-lateral width of head	MT1-H-MLW	17.40	0.611	3.5

MEAN = mean of the means obtained by the two independent observers; TEM = absolute technical error of measurement; RTEM = relative technical error of measurement (TEM*100/MEAN). Figures in bold = error value not acceptable.

Measurements were taken in mm by two independent observers using a digital caliper; the technical error of measurement (TEM) and the relative technical error of measurement (RTEM) were calculated (Table 2) [68].

F and Bartlett’s tests were used to evaluate the null hypothesis of no difference between the variances of the traits in males and females [69]. For each variable, mean and standard deviation were calculated and a t-test for independent samples was run to verify the statistical difference between the means for archaeological males and females. The estimation of sex dimorphism was carried out using the approach of Chakraborty and Majumder [70], which calculates the areas of non-overlap (D) in the two normal distributions, derived from the means and standard deviations by sex for each trait and the cut-off point (x0) for sex estimation (S1 Appendix).

A cross-validation approach was adopted in order to validate the discriminatory power of each trait [71]. Through this resampling method it was possible to estimate, in a consistent way, the accuracy and the precision of each metric trait in the sex assessment.

The cross-validation analysis was run for each trait by: (1) random selection of a training set of individuals, corresponding to around 70% of the whole sample, with both genders equally represented; the remaining 30% of the individuals formed the test set, to be classified; (2) calculation, for the training set, of the Chakraborty and Majumder D and the cut-off point for the anatomical trait; (3) sex classification of the test set, according to the cut-off point determined for the training set; (4) creation of the 2x2 confusion matrix as follows: the cell in row 1 and column 1 (CA) represents the number of archaeological females correctly classified as females; the cell in row 2 and column 2 (CD) represents the number of archaeological males who are correctly classified as males; the cell in row 1 and column 2 (CB) represents the number of archaeological females who are incorrectly classified as males; and the cell in row 2 and column 1 (CC) represents the number of archaeological males who are incorrectly classified as females; (5) calculation, from the confusion matrix, of the accuracy of sex determination as the sum of CA plus CC divided by the total number of individuals in the test set; (6) calculation, from the confusion matrix, of the precision of sex determination for females as CA divided by the number of females in the test set; (7) calculation, from the confusion matrix, of the precision of sex determination for males, as CA divided by number of males in the test set; (8) repetition of steps 1 to 6 1000 times, and calculation of the mean and the standard deviation for accuracy, precisions and cut-off points.

The analyses were performed with the R language and environment for statistical computing version 3.4.2 [72] and the caret package [73] for the test/training partitions of the dataset.

## Results

Results for the inter-observer error estimate are reported in Table 2. All variables, with three exceptions, show a RTEM (relative index of inter-observer differences) below the acceptance threshold of 0.05. The three variables showing an unacceptable level of error are the height of the dens of the axis (AX-D-H), the humeral head transverse diameter (HU-H-TD), and the mandible condyle thickness (MAN-C-TH). These were therefore excluded from the subsequent analyses.

Table 3 presents the descriptive statistics for each metric variable and the results of the Chakraborty and Majumder test ([70]; D-value), cut-off points and t-test.

**Table 3:** Descriptive and inferential statistics of 21 variables.

*Trait*	*Abbreviation*	*Males N*	*Females N*	*Males mean* *(mm)*	*Females mean* *(mm)*	*Males s*.*d*. *(mm)*	*Females s*.*d*. *(mm)*	*Student’s t*	*t probability*	*Degree of freedom*	*D value*	*D sd*	*cut-off point* *(mm)*
Mandible: condyle width	MA-C-W	17	17	17.15	14.66	1.38	1.60	-4.87	<0.001	31.29	0.60	0.14	15.87
Axis: dens anteroposterior diameter	AX-D-APD	27	20	10.00	9.04	0.88	0.83	-3.82	<0.001	42.25	0.43	0.13	9.55
Axis: dens transverse diameter	AX-D-TD	26	20	9.08	8.82	0.79	0.74	-1.18	0.24	42.35	0.14	0.14	9.10
Humerus: vertical head diameter	HU-H-VD	10	15	40.37	35.42	2.52	2.26	-5.01	<0.001	17.88	0.70	0.15	37.88
Humerus: trochlea maximum diameter	HU-T-MXD	13	18	20.91	18.81	1.75	1.37	-3.61	<0.001	21.84	0.51	0.16	20.00
Humerus: trochlea minimum diameter	HU-T-MID	25	31	13.85	12.23	1.69	1.28	-3.96	<0.001	43.96	0.43	0.12	13.28
Humerus: capitolum maximum diameter	HU-C-MXD	12	14	17.07	15.03	1.33	1.13	-4.19	<0.001	21.81	0.60	0.16	16.09
Radius: head maximum diameter	RD-H-MD	26	34	19.76	16.91	1.31	1.21	-8.62	<0.001	51.70	0.74	0.09	18.32
Lunate: max width	LU-MXW	8	8	15.32	13.29	0.91	1.18	-3.84	<0.001	13.15	0.67	0.18	14.30
Lunate: max length	LU-MXL	6	11	14.83	12.79	0.81	1.24	-4.10	<0.001	14.27	0.69	0.17	13.82
Femur: vertical head diameter	FE-H-VD	10	15	42.10	36.60	3.31	3.06	-4.19	<0.001	18.33	0.61	0.16	39.39
Patella: maximum height	PA-MXH	7	12	37.22	33.51	3.30	2.57	-2.56	0.03	10.30	0.48	0.21	35.68
Patella: maximum width	PA-MXW	8	13	38.92	34.59	2.37	1.47	-4.65	<0.001	10.38	0.75	0.15	36.61
Patella: maximum thickness	PA-MXTH	22	38	16.67	14.70	2.32	1.71	-3.48	<0.001	34.35	0.39	0.12	16.10
Talus: maximum length	TA-TL	6	11	48.84	44.93	2.41	2.50	-3.15	0.01	10.72	0.57	0.21	46.87
Talus: head-neck length	TA-HN-L	8	12	18.76	17.54	2.71	3.68	-0.85	0.40	17.73	0.20	0.21	16.51
Talus: trochlea length	TA-TR-L	10	14	31.14	26.68	2.01	2.27	-5.07	<0.001	20.85	0.70	0.15	28.92
Talus: trochlea width	TA-TR-W	18	29	28.99	25.72	2.71	2.35	-4.22	<0.001	32.22	0.48	0.13	27.52
Navicular: maximum lenght	NA-MXL	15	23	14.00	11.94	2.46	1.61	-2.86	0.01	21.86	0.41	0.15	13.46
First metatarsal: dorso-plantar width of the head	MT1-H-DPW	23	31	17.14	15.13	1.41	1.29	-5.38	<0.001	45.14	0.54	0.12	16.17
First metatarsal: medio-lateral width of the head	MT1-H- MLW	17	21	18.19	15.93	1.32	1.52	-4.89	<0.001	35.76	0.57	0.13	17.02

Chakraborty and Majumder’s D-values, standard deviation of D-values, cut-off points.

Sample size is highly variable across the metrical traits, with differences between sexes: the average number of observations is 15 for males and 19.4 for females.

Results of the F and Bartlett’s tests show that for all the variables the differences between the male and female variance are not statistically significant (p>0.05).

Student’s t-test displays high significance for all traits (p<0.05), except the dens transverse diameter of axis and head-neck length of talus, with a *p*-value of 0.24 and 0.40 respectively.

**Fig 5:**
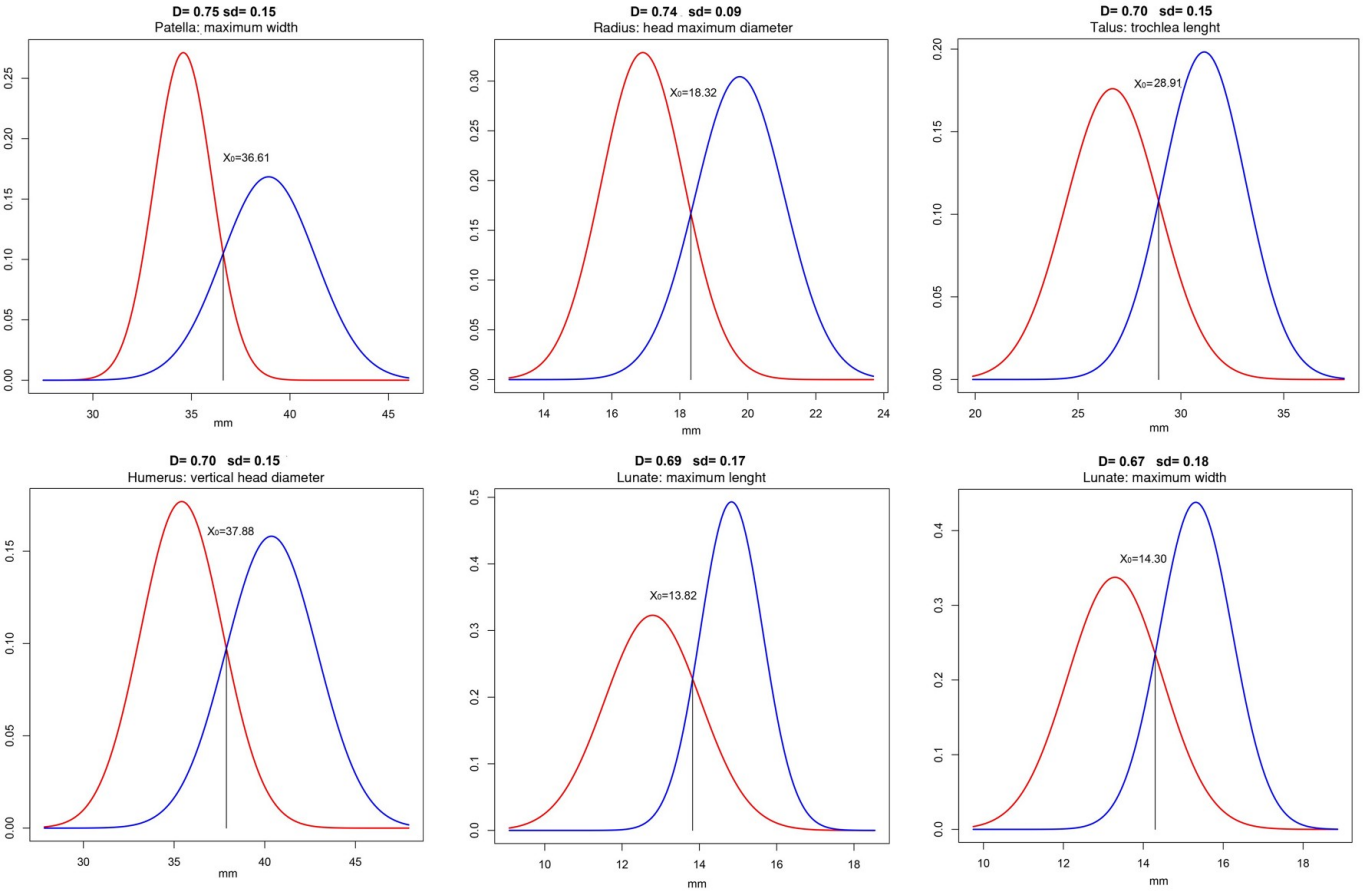
The most dimorphic traits. Normal distribution curves derived from the sample female (red line) and male (blue line) means, standard deviations and N. D = Chakraborty and Majumder’s D-value; sd = standard deviation; X0 = cut-off point.

Chakraborty and Majumder’s D-values (non-overlapping area of the male and female distributions) appear very high (D>0.70) in four cases: patella maximum width (D_PA-MXW_ = 0.750); radius head diameter (D_RD-H-MD_ = 0.741); talus trochlea length (D_TA-TR-L_ = 0.703) and humeral head vertical diameter (D_HU-H-VD_ = 0.701). D-values superior to 0.6 are shown by the maximum length and width of the lunate (D_LU-MXL_ = 0.692 and D_LU-MXW_ = 0.671) and the vertical diameter of the femoral head (D_FE-HVD_ = 0.613) (Fig 5). Low values of non-overlapping area (D<0.4) are registered for the transverse diameter of the dens of axis (D_AX-D-TD_ = 0.14), the head-neck length of talus (D_TA-HN-L_ = 0.20), and the maximum thickness of patella (D_PA-MXTH_ = 0.39).

Table 4 reports the accuracy and precision for the traits as derived from the cross-validation analysis. Eight variables out of 21 show an accuracy (concordance between estimated skeletal sex and archaeological gender) that exceeds or equals 80%, so that, using the calculated cut-off points, a mean of 8 out of 10 individuals is expected to be accurately classified as male or female. These variables are: radius head diameter (88.3% of accurate determinations), patella maximum width (86.0%), mandibular condyle width (83.5%), talus trochlea length (83.2%), femural head vertical diameter (81.3%), dorso-plantar width of the head of the first metatarsal (80.6%), lunate length (80.2%), humeral head vertical diameter (80.0%). Radius head diameter is certainly the most dimorphic trait, as the potential of misclassification is below 12% for both sexes with a cut-off point of 18.32 mm. A further nine variables show an acceptable percentage of accuracy (higher than 70%), while the remaining four traits are unreliable (values between 48.5% and 69.2%).

**Table 4:** Cross-validation results in sex estimates.

*Trait*	*Abbreviation*	*Accuracy* *(%)*	*Accuracy s*.*d*.	*Precision in male diagnosis* *(%)*	*Precision in female diagnosis* *(%)*	*Training set N*	*Test set N*	*Cut-off mean* *(mm)*	*Cut-off s*.*d*. *(mm)*
Radius: head maximum diameter	RA-H-MXD	88.3	6.7	88.1	88.5	43	17	18.32	0.12
Patella: maximum width	PA-MXW	86.0	13.1	75.5	93.0	16	5	36.61	0.22
Mandible: condyle width	MA-C-W	83.5	10.2	83.9	83.2	24	10	15.87	0.19
Talus: troclea length	TA-TR-L	83.2	12.0	79.1	86.3	17	7	28.91	0.31
Femur: vertical head diameter	FE-H-VD	81.3	12.9	82.7	80.2	18	7	39.42	0.45
First metatarsal: medio-lateral width of the head	MT1-H-MLW	80.6	9.9	82.3	79.1	27	11	17.03	0.16
Lunate: maximum length	LU-MXL	80.2	18.4	84.8	78.6	13	4	13.82	0.13
Humerus: vertical head diameter	HU-H-VD	80.0	13.1	73.4	84.1	18	7	37.88	0.32
Humerus: capitolum maximum diameter	HU-C-MXD	78.9	13.7	73.5	82.9	19	7	16.09	0.16
Lunate: maximum width	LU-MXW	78.3	19.4	82.1	74.5	12	4	14.30	0.18
Talus: maximum length	TA-TL	78.2	18.6	68.6	81.4	13	4	46.81	0.38
First metatarsal: dorso-plantar width of the head	MT1-H-DPW	75.6	9.4	71.4	78.4	39	15	16.17	0.13
Navicular: maximum length	NA-MXL	74.6	12.4	63.6	81.9	28	10	13.46	0.22
Patella: maximum thickness	PA-MXTH	74.0	8.7	62.6	80.3	43	17	16.10	0.18
Humerus: trochlea maximum diameter	HU-TR-MXD	73.1	13.4	70.1	74.9	23	8	19.98	0.27
Humerus: trochlea minimum diameter	HU-TR-MID	72.3	9.5	63.0	79.5	40	16	13.27	0.16
Talus: trochlea width	TA-TR-W	71.7	11.3	66.0	75.4	34	13	27.49	0.32
Patella: maximum height	PA-MXH	69.2	17.4	62.1	74.0	14	5	35.57	0.69
Axis: dens anteroposterior diameter	AX-D-APD	67.8	10.8	68.8	66.5	33	14	9.55	0.14
Axis: dens transverse diameter	AX-D-TD	56.5	10.2	46.3	68.3	33	13	9.04	0.33
Talus: head-neck length	TA-HN-L	48.5	17.1	79.9	27.5	15	5	16.88	1.69

The variables are listed according to the decreasing accuracy level. For all traits, the resampling simulation was run 1000 times.

Some measurements taken on the same bone show distinct levels of discriminatory power. Patella width and thickness seem to be a reliable discriminant (PA-MXTH accuracy = 74.0% and PA-MXW accuracy = 86.0%), while height is less so (PA-MXH accuracy = 69.2%); the dens anteroposterior diameter of axis shows a higher accuracy (67.8%) than the transverse diameter (56.5%).

The precision in sex estimation for each single variable is generally greater for females (14 traits) than for males (7 traits).

## Discussion

The aim of this study was to test the applicability of univariate metric techniques for sex diagnosis of cremated individuals. From the initial set of 24 variables selected, three were subsequently excluded from the analysis due to an unacceptable level of error at the TEM calculation. Overall, the results demonstrated that ancient calcined bones can preserve a good degree of sexual dimorphism that is not biased (or only minimally) by the augmentation or shrinkage of the heating process, as already reported by previous studies [35–42,44,53].

Eight of the 21 analyzed variables showed a degree of accuracy in the sex assessment that was equal to or greater than 80%, a value generally considered a benchmark for evaluating the utility of a determination method [74,75]. The most discriminatory measurements are located on the radius, patella, mandible, talus, femur, first metatarsal, lunate and humerus. Radius head diameter is the most dimorphic trait, as the potential of correct classification–with a cut-off point of 18.32 mm–is 88.1% for the males, 88.5% for the females and 88.3% for pooled sexes.

When comparing the discriminating power of the present analysis with results offered for the same measurements on unburned modern series of known sex, the estimates are broadly of the same order (Table 5) with few exceptions. Within the 13 variables compared, 10 present an accuracy level very close to or even exceeding those reported by other studies. This is the case for the radius head. A study by Barrier and L’Abbé [76] on a reference collection of 400 unburned individuals of known sex obtained an accuracy of 80.7%. In the study by Berrizbeitia [77], for the same measurement, sex is correctly identified for 83% of the sample, but this method included a 3 mm non-diagnosis interval (from 21 to 23 mm). The patella maximum width (the second most dimorphic variable in our series) yields 86.0% of correct diagnoses, a value far exceeding other estimates [78–80]. Three variables perform worse than the unburnt comparative samples: the humeral head vertical diameter, the lunate maximum width and the talus head-neck length.

**Table 5:** Sex estimation accuracy levels registered in the present study and studies on unburned modern samples.

*Trait*	*Study*	*Accuracy*
**Humerus: vertical head diameter**	**Present study**	**80.0%**
	[86]	90.4%
	[87]	89.9%
	[88]	89.9% (L), 91.4% (R)
**Radius: head maximum diameter**	**Present study**	**88.3%**
	[86]	88.6%
	[89]	82.0%
	[90]	83.0%*
	[88]	94.6% (L), 94.1% (R)
**Lunate: maximum length**	**Present study**	**80.2%**
	[91]	83.5%
**Lunate: maximum width**	**Present study**	**78.3%**
	[91]	90.1%
**Femur: vertical head diameter**	**Present study**	**81.3%**
	[92]	86.8%
	[93]	82.3%
	[94]	94.8%
	[95]	85.9%
	[96]	76–93.5%**
**Patella: maximum width**	**Present study**	**86.0%**
	[97]	72.5%
	[98]	76.9%
	[99]	79.2%
**Patella: maximum thickness**	**Present study**	**74.0%**
	[97]	86.0%
	[98]	76.9%
	[99]	75.8%
**Patella: maximum height**	**Present study**	**69.2%**
	[97]	71.3%
	[98]	80.8%
	[99]	85.0%
**Navicular: maximum length**	**Present study**	74.6%
	[100]	73.4% (L), 76.4% (R)
**Talus: maximum length**	**Present study**	**78.2%**
	[101]	81.7%
	[102]	81.0%
	[103]	84.0%
	[100]	84.4% (L), 90.0% (R)
**Talus: trochlea length**	**Present study**	**83.2%**
	[101]	71.7%
**Talus: trochlea width**	**Present study**	**71.7%**
	[101]	74.2%
**Talus: head-neck length**	**Present study**	**48.5%**
	[101]	73.3%

* the method includes a 3 mm of non-diagnosis interval;

** range of 4 South African white series.

For comparison with cremated samples, we must currently rely on only the study of Gonçalves et al. [53] on contemporary Portuguese cremated individuals and the study of Van Vark on contemporary Swedish [38].

The three variables used by Gonçalves (and by our own study) are the vertical head diameters of humerus and femur and the maximum length of the talus. As shown in Table 6, the Late Bronze Age/Iron Age Italian series regularly show lower mean values for both sexes; the differences range from -0.64 mm (maximum length of the talus in the females) to -3.14 mm (humerus vertical head diameter in males), plausibly a consequence of a difference in the body mass between diverse populations. An even greater difference exists with the two traiys measured by Van Vark.

**Table 6:** Sexual dimorphism and correct sex classification.

Present study										
*Trait*	*Males N*	*Females N*	*Males mean* *(mm)*	*Females mean* *(mm)*	*Males s*.*d*. *(mm)*	*Females s*.*d*.	*D value*	*Precision in male diagnosis*	*Precision in female diagnosis*	*Accuracy*
Humerus: head vertical diameter	10	15	40.37	35.42	2.52	2.26	0.70	73.4%	84.1%	80.0%
Femur: head vertical diameter	10	15	42.10	36.60	3.31	3.06	0.61	82.7%	80.2%	81.3%
Talus: maximum length	6	11	48.84	44.93	2.41	2.50	0.57	68.6%	81.4%	78.2%
[53]										
*Trait*	*Males N*	*Females N*	*Males mean* *(mm)*	*Females mean* *(mm)*	*Males s*.*d*. *(mm)*	*Females s*.*d*. *(mm)*	*D value*	*Precision in male diagnosis*	*Precision in female diagnosis*	*Accuracy*
Humerus: head vertical diameter	62	62	43.51	37.74	2.89	2.98	0.67	86.0%	100%	88.1%
Femur: head vertical diameter	55	55	43.02	37.64	3.34	2.18	0.68	87.5%	90.0%	87.5%
Talus: maximum length	30	30	50.97	45.57	3.15	2.93	0.63	75.0%	100%	75.8%
[38]										
*Trait*	*Males N*	*Females N*	*Males mean* *(mm)*	*Females mean* *(mm)*	*Males s*.*d*. *(mm)*	*Females s*.*d*. *(mm)*	*D value*	*Precision in male diagnosis*	*Precision in female diagnosis*	*Accuracy*
Humerus: head vertical diameter	103	108	44.9	38.7	2.7	2.7	0.75	88%	88%	87%
Femur: head vertical diameter	104	108	45.9	39.9	2.3	2.7	0.77	91%	88%	90%

Comparison between a contemporary cremated series [53] and data from the present study on three metrical variables. D value = Chakraborty and Majumder’s calculation.

The indexes of sexual dimorphism (D values) and the reliability of sexual estimate are slightly lower for the protohistoric sample. Nevertheless, the application of the modern series cut-off points (recalculated through the Chakraborty and Majumder’s method) to our ancient samples (Table 7) clearly yields lower percentages of correct classifications for the humerus head diameter (50% of misclassification in the males using Gonçalves et al.’s method and even 90% with Van Vark’s) and for the femur head diameter (30% of misclassification in the males using Gonçalves et al.’s method and 60% with Van Vark’s). By contrast, the 33.3% level of incorrect diagnosis for the talus applying Gonçalves et al.’s method is very close to the result obtained by the cross-validation analysis of the ancient sample (see also Table 6). Overall, the results suggest caution in applying standards based on contemporary specimens—whose value is nonetheless unquestioned for forensic cases and relatively modern populations—to chronologically and geographically distinct samples, and reinforces the need to build population-specific standards.

**Table 7:** Application of the cut-off point calculated from Gonçalves et al. [53] and Van Vark [38] to the present study series.

*Trait*	*Study*	*Precision in male diagnosis*	*Precision in female diagnosis*	*Accuracy*
Humerus: head vertical diameter	[53]	50%	100%	80%
	[38]	10%	100%	64%
Femur: head vertical diameter	[53]	70%	80%	76%
	[38]	40%	100%	76%
Talus: maximum length	[53]	66.7%	81.8%	76.5%

This study contributes to the debate on the extraction of demographic profiles from cremated human remains [81–83]. The unpredictable and extremely variable number and nature of observable traits in the cremains has been seen as a limitation in standardizing analytical procedures, minimizing the reproducibility and comparability between different contexts and researchers. Indeed, the majority of anthropological contributions to archaeological research are insufficiently detailed to provide a clear understanding of the methods applied in sex assessment (see for instance [84]), and are frequently relegated to a brief “osteological appendix”. In this respect, Gonçalves and Pires [85] conducted a survey on the consistency of approaches and methodologies used among researchers in the analysis of cremation contexts. On a sample of 84 published papers, 95% reported individual sex assessments, but fewer than 30% had applied metric methods. The Authors claim that osteometry on cremains is still fundamentally mistrusted by researchers, given the fact that metrical criteria specifically developed from/for cremated remains are scant [35,42,43,53]. Our study has proved that the osteometric approach is indeed a feasible approach and should be further investigated and applied in archaeological contexts. Moreover, this method can be used on very fragmented individuals, when morphologies are not easily readable. Its application can yield better results when considered together with other sex indicators, such as the morphological traits of skull and pelvis.

The initial assumption that gender is the only viable proxy for sex (necessarily unknown in prehistoric cremated populations) should not be seen as a limitation but, instead, as an opportunity. From a purely interdisciplinary perspective, this method (or any further development of it), would enable the detection of outliers, namely unusually robust individuals with typical feminine grave good assemblages or, vice-versa, unusually gracile individuals archaeologically characterized as men. For these individuals estimated sex and gender might not coincide and may need further discussion. Without any common, replicable metrical base, researchers may abandon ambitions to explore the relationships between sex and gender and the socio-demographic dynamics of Bronze Age and Iron Age populations.

Despite our cremated collection comprises different populations, both in terms of chronology and geography, this diversity does not seem to affect negatively our results and the good degree of sexual dimorphism encourages further development of the osteometric technique. We might assume that if larger homogeneous samples were available, results could have been even more significant.

## Conclusions

Gonçalves and Pires assert that, “if bioarchaeologists hope to approach broad crosscultural themes and simultaneously understand the chronological and geographical diversity of cremation-related funerary practices […], they need to rethink and standardize their procedures” [85]. In this vein, the present study begins the task of building new osteometric methods for the sex estimation of ancient human cremated remains, using 124 Late Bronze Age and Iron Age adult individuals from Italy, whose gender is clearly indicated by grave goods.

Our results demonstrate that the most dimorphic traits are located on the epiphysis of the long bones, carpal and tarsal bones, the first metatarsal, patella, and mandibular condyle, and that the accuracy of diagnoses is broadly similar to those obtained on unburnt series of known sex, with few exceptions. The mean values and cut-off points are significantly lower than those for modern cremated samples. Nevertheless, indexes of sexual dimorphism show the same degree of male/female dimensional difference, making us more confident about estimating the sex from human cremated remains.

As long as sample sizes allow it, the use of population-specific metric references appears to be a useful procedure to provide more objective and reproducible sex attributions, or in cases where grave goods are completely absent. This approach cannot be used in small samples though. For such cases, data obtained by this research may eventually be used as reference. The next step is to validate these references on analyses involving other pre- and protohistoric European skeletal series. In the future, we intend to enlarge our sample size, and the number of metric traits (especially on carpals and tarsals), but also trying to develop multivariable approaches that could reinforce the accuracy of sex estimation and clarify the relationship between sex and gender on a more objective basis.

## Supporting information

S1 Table List of burials and measurements. Cells marked in blue indicate that the trait is “masculine” compared to the cut-off point (x_0_); cells marked in pink indicate that the trait is “feminine” compared to the cut-off point.
(XLSX)

S1 Appendix ‘R-statistics’ function to calculate the Chakraborty Majumber index of sexual dimorphism of metric traits. (R)
